# Knowledge, attitude, practice and prevalence of traditional cauterization among patients in Massawa Hospital, Eritrea: a cross-sectional study

**DOI:** 10.11604/pamj.2020.36.230.21349

**Published:** 2020-07-29

**Authors:** Berhe Tesfai, Adhanom Debesai, Saliem Mekonnen, Fnan Girmay, Fitsum Kibreab, Lemlem Hussien, Mulugeta Russom

**Affiliations:** 1 Massawa Hospital, Northern Red Sea Zone, Massawa, Eritrea,; 2 Health Research and Resources Centre Division, Ministry of Health, Asmara, Eritrea,; 3 National Medicines and Food Administration, Ministry of Health, Asmara, Eritrea

**Keywords:** Traditional cauterization, knowledge, attitude

## Abstract

**Introduction:**

use of traditional cautery for the treatment of varied ailments is one of the most ancient and harmful traditional medical practices that is still in use. This study was conducted to assess the knowledge, attitude, practice and to estimate the prevalence of traditional cautery among patients visiting Massawa hospital.

**Methods:**

a hospital-based cross-sectional study was conducted in Massawa hospital from February 20 to April 20, 2019. The study enrolled all patients aged >18 years, non-critical and willing to participate.

**Results:**

a total of 900 participants were enrolled in the study. The study was dominated by Muslims (81.4%) with a median age of 42 years (IQR: 20). Self-reported prevalence of traditional cauterization was found to be 43.6% and 63% of them did their latest cautery between 2011 and 2019. Metal rods were used as cauterant in 92.3% and 47.9% reported that traditional practitioners used the same cauterant for different people. One-third of the respondents reported that it is a safe practice and 47% had the understanding that it cannot transmit communicable diseases. Moreover, 90.4% of the study participants reported that they knew someone who did cautery. Respondents with poor knowledge (AOR=6.45, 95% CI: 4.69-8.87) and attitude (AOR=8.68, 95% CI: 6.31-11.95) were more likely to practice cauterization compared to those with good knowledge and attitude.

**Conclusion:**

the practice of cauterization in visitors of Massawa hospital was rampant which is mainly associated with poor knowledge and attitude of the respondents, limited access to health facilities and religious/cultural conviction.

## Introduction

Traditional cauterization is defined as the application of hot metal rods/knives, and/or fire over diseased body parts for therapeutic purposes [1,2]. The metal rods/sticks are heated and practitioners place the hot metal over the affected skin for a few seconds [3-6] and the number of cauterization per one session varies between one to seven [5]. Though the choice of location for cauterization depends on the disease condition of patients and other factors, the whole body, including the face and head, is at risk of the practice [3,4].

Even though traditional practitioners believe that cautery prevent or treat diseases, current studies revealed that it causes severe infections and complications as adverse effect such as septic shock, cavernous sinus thrombosis, multiple splenic abscesses, deep scars, potentially chronic serious diseases, permanent unilateral blindness, physical deformities and even death [3,4,7]. Despite its unbearable adverse outcomes, significant portion of the population living in the developing countries practice traditional cautery [3,4,8,9]. In Somalia, cautery was the main therapy for alleviating various diseases such as hepatitis, facial paralysis, parotitis, and childhood rickets and almost all Somalis are reported to be cauterized in their lifetime with wood, palm leaves or iron rode [3,4].

Although the prevalence of traditional cautery among the Eritrean population is unknown, in the last few years, among patients in Massawa hospital, several patients had visited either with complications and/or scars of cauterization in their body. Based on the hospital records and experience, it was the authors´ assumption that the practice is prevalent among all ethnic groups residing in Massawa sub-zone. As a result this study was aimed to assess the knowledge, attitude, practice and measure the prevalence of traditional cauterization among Massawa hospital visitors between February and April 2019.

## Methods

**Study design and setting**: a cross-sectional hospital-based study was conducted in Massawa hospital, Eritrea. Massawa hospital is found in the city of Massawa; about 102 kilometers towards North-East of Asmara, the capital city of Eritrea.

**Source and study population**: Massawa hospital is the only hospital in the sub-zone and provides services to general public living in the sub-zone and beyond either referred or self-referred from different parts of the nearby sub-zones. Thus, as of 2019, the source population of this study is estimated to be above 44,815 (source: Massawa subzone administration, 2018). The Sub-Zone of Massawa includes five of the nine ethnic groups available in Eritrea. All patients visiting Massawa hospital during the study period aged 18 years and above and consented to participate were enrolled in the study. For patients with multiple hospital visits during the study period, they were considered only once. The pediatric population were excluded from the study for ethical and possible lack of clarity of response to fill the questionnaire. Moreover, patients visiting the hospital with a serious illness and/or with some level of deranged consciousness and with mental illness were also excluded from the study for appropriateness to fill the questionnaire.

**Data collection tools and approach**: data was collected from February 20 to April 20, 2019. A structured questionnaire which was modified from a previous similar study entitled “traditional cautery for medical treatment among the Bedouins of Southern Israel” [8], was used as a data collection tool after efforts were made to adopt it to the local context and to the objectives of the study. The questionnaire had two main parts, the socio-demographic characteristics and the knowledge, attitude and practice variables. Socio-demographic characteristics including access to health facilities, the need for means of transport and their availability and several questions that test the knowledge, belief and practice of the study population were included in the questionnaire [questionnaire is attached as supplemental digital content]. The questionnaire was filled through exit interview for out-patients and for those who were admitted to adult wards of the hospital during the study period. The selected data collectors were linguists who know majority of the languages of the sub-zone and trained on how to conduct the data collection process prior to commencement of the study. To ensure quality of the data collection, there was intensive supervision by the principal investigators.

**Data measurement and interpretation**: study participants´ knowledge on traditional cauterization was assessed using different parameters (questions). Each parameter was awarded 1 mark for the correct answer and 0 mark if the answer was wrong. Those who scored 50% and above were considered as having adequate knowledge, and those who scored below 50% were considered as having inadequate knowledge. Variables to assess attitude included the participants view on traditional cauterization. Each question was awarded 1 for correct response and 0 otherwise. Those who scored equal to and above 50% were considered as having positive attitude. Their practice was also assessed as each parameter was awarded 1 mark for good practice and 0 mark for inappropriate practices. Those who scored 50% and above were considered as practicing adequately and those who scored below 50% were considered as inadequate practices with regard to traditional cauterization.

**Analysis**: data was entered through CSPro and analyzed by SPSS version 20. Both descriptive and analytical statistics were performed. Frequency, percentage, with standard deviation and odds ratio with 95% confidence interval was used as a measure of association and statistical significance was tested at p-value <0.05.

**Ethical consideration**: ethical clearance to conduct this study was obtained from the Ministry of Health research ethical and protocol review committee. Approval was also achieved from the Ministry of Health of Northern Red Sea branch and from the director of Massawa hospital. Prior to data collection, written consent was obtained from every study participant and personal identifiers were coded and anonymized while entered into CSPro to protect patients´ confidentiality.

## Results

**Socio-demographic characteristics of the study population**: the study enrolled a total of 900 participants with majority of them being males (56%) and the median age was 42 years (interquartile range: 20). The composition of the respondents´ ethnicity was Tigre (30.9%), Afar (23.0%), Saho (20.0%), Tigrigna (19.3%) and others (0.7%). Majority of the respondents (66.4%) were from Massawa sub-zone and they were dominated by Muslims (81.4%). In regard to their marital status, more than three fourth (78.9%) were married followed by single (15%) and divorced (3.2%). Educationally, almost 47% of the respondents have never had any formal education; while others attended primary school (19.9%), middle school (14.1%), high school (9.9%) and higher education (9.2%). Furthermore, almost 79% reported that they don´t have any known chronic diseases. Significant number of the study participants (94.6%) reported that primary healthcare services were either accessible (43.4%) or moderately accessible (51.2%) to them. The rest 5.4% reported that it was inaccessible. Though most of the respondents reported that primary healthcare service was easily or moderately accessible, 91.7% required means of transport to visit health facilities. And, 45% of the study participants reported that means of transportation to their respective health facilities was easily accessible. However, 55% of respondents stated that means of transport was either hardly available (48.5%) or not at all (6.5%). Seventy-three percent of the respondents also have access to healthcare services within 10 kilometers. Considering their monthly income, respondents informed that 40.6% make less than 500 ERN, 39% earn 500-1000 ERN and 20.4% above 1000 ERN (Table 1).

**Table 1:** socio-demographic characteristics of the study participants

Variables		Frequency (N)	Percent (%)
**Age**	Less than 20	12	1.3
	20-39	359	39.9
	40-59	422	46.9
	60 and above	107	11.9
**Sex**	Male	504	56
	Female	396	44
**Religion**	Christian	167	18.6
	Muslim	733	81.4
	Housewife	208	23.1
**Main occupation**	Government employee	181	20.1
	Farmer	167	18.6
	Unemployed	140	15.6
	Fisherman	89	9.9
	Private employee	56	6.2
	Self employed	53	5.9
	Other	6	0.7
**History of chronic disease**	Yes	176	19.6
	No	707	78.6
	Don't know	17	1.9
**Accessibility to a primary healthcare service**	Accessible	391	43.4
	Moderately accessible	460	51.1
	Not accessible	49	5.4
**Required transportation to the primary health facility**	Yes	825	91.7
	No	75	8.3
**Availability of means of transportation**	Easily available	405	45.1
	Limited access	436	48.5
	Not available	58	6.5
**Total**		900	100

**Knowledge and Attitude of the study participants on traditional cauterization**: almost 95% of the respondents have heard about traditional cauterization in the past. Of respondents, 41.1% had the understanding that traditional cauterization was not safe; while 33.7% admitted that it is a safe practice. The rest, 25.2%, did not know about the safety of traditional cauterization. Almost 47% of the respondents mentioned that traditional cauterization cannot transmit communicable diseases like HIV and hepatitis. About half (45.9%) of the respondents had the conception that traditional cauterization cured and 42.9% reported that it could not cure illnesses. Majority of the respondents (81.1%) were either wholly (44.6%) or partially (36.5%) satisfied about their treatment with cautery while the rest were not satisfied. One-third of the participants (33.4%) supported the practice of cauterization by the general public. Of the respondents, 34.3% had a belief that traditional healers were knowledgeable and 42.4% reported that they were not. Moreover, the main reasons for their use of traditional cautery were to treat incurable conditions by the conventional medicine (60.8%) followed by the belief that the practice was more effective and safe (38.6%) (Table 2). The participants were also asked to provide their opinion regarding stopping traditional cauterization practices and almost 29% of the respondents did not support the termination of the practice; while 55.6% agreed that it should be stopped (Table 2).

**Table 2:** knowledge, attitude and practice of study participants on traditional cauterization

Question	Response	Frequency (N)	Percent (%)
**Cauterization is generally safe**	Agree	289	33.6
	Neutral	216	25.2
	Disagree	353	41.2
**Do you think cauterization cures?**	Yes	394	45.9
	No	368	42.9
	Don't know	96	11.2
**Traditional cauterization can transmit communicable diseases like HIV**	Agree	239	26.5
	Neutral	199	22.1
	Disagree	420	46.6
**Do you agree with your community's support of cauterization?**	Agree	287	33.4
	Neutral	202	23.5
	Disagree	369	43.0
**Do you plan to use traditional cautery in the future?**	Yes	156	18.2
	No	603	70.4
	Don't know	98	11.4
**Do you think there are diseases that can be cured by cautery?**	Yes	523	61.0
	No	274	31.9
	Don't know	61	7.1
**Do you believe traditional cautery practice should be stopped?**	Agree	460	53.6
	Neutral	152	17.7
	Disagree	246	28.7
**Tools used for traditional cauterization?**	Metal rod	831	92.3
	Wood/thorn	104	11.6
	Knife	66	7.3
	Don't know	16	1.8
**Did the traditional healers use same cauterant?**	Yes	411	47.9
	No	125	14.6
	Don't know	322	37.5
**Did they sterilize the cauterant after the procedure?**	Yes	402	46.9
	No	62	7.2
	Don't know	394	45.9
**Tools used for sterilization**	Fire	271	66.9
	Boiled water	120	29.6
	Others (sunlight, rinsing with water)	14	3.4

**Prevalence and practice of respondents on traditional cauterization**: of the study participants, 45.7% had practiced traditional cautery at least once in their life. Meanwhile, 73.5% of the respondents don´t encourage others to practice cauterization; while 26.5% had been encouraging others. About three-fourth (75.8%) of the study participants disclosed that they didn´t inform healthcare professionals about their history of traditional cautery, during their visit to health facilities. On their use of traditional cautery, however, they mainly followed advises from families (38.6%), followed by relatives (23.4%). Metal rods were used for traditional cauterization in 92.3% of the cases and about half (47.9%) of the respondents mentioned that traditional medical practitioners used the same cauterant for different people during their session. About half of the respondents (47.9%) also reported that they sterilized the cauterants mainly with fire (66.9%) followed by boiled water (29.6%) (Table 3).

**Table 3:** association of socio-demographic characteristics, and comprehensive knowledge, attitude and practice with prevalence of cautery

Demographic characteristics		Have you ever had traditional cautery in your life? N=858		p-value	Odds ratio (95% CI)
Age group	Count	Yes (%)	No (%)		
18-39	345	37.4	62.6	<0.001	1
40-59	407	49.6	50.4		1.65 (1.23 - 2.21)
60 and above	106	57.5	42.5		2.27 (1.46 - 3.53)
**Sex**					
Male	472	45.1	54.9	0.716	1
Female	386	46.4	53.6		1.05 (0.8 - 1.38)
**Religion**					
Christian	134	8.2	91.8	<0.001	1
Muslim	724	52.6	47.4		12.42 (6.59 - 23.41)
**Education**					
No education	420	59.5	40.5	<0.001	1
Primary	178	44.4	55.6		0.54 (0.38 - 0.77)
Middle & above	260	45.7	54.3		0.22 (0.15 - 0.31)
**Marital status**					
Single	127	29.1	70.9	<0.001	1
Ever married	731	48.6	51.4		2.3 (1.53 - 3.46)
**Access to health facility**					
Accessible	353	30.9	69.1	<0.001	1
Moderately accessible	456	53.9	46.1		2.62(1.96-3.51
Not Accessible	49	75.5	24.5		6.90(3.46-13.75)
**Ethnicity**					
Tigrigna	139	6.5	93.5	<0.001	1
Afar	206	55.3	44.7		17.90(8.63-37.11)
Tigre	273	50.5	49.5		14.77(7.22-30.21)
Saho	180	50.6	49.4		14.77(7.07-30.83)
Rashaida	54	68.5	31.5		31.44(12.95-76.30)
**Comprehensive knowledge**					
Good knowledge	339	20.4	79.6	<0.001	1
Poor knowledge	519	62.2	37.8		6.45 (4.69 - 8.87)
**Comprehensive attitude**					
Good attitude	383	19.1	80.9	<0.001	1
Poor attitude	475	67.2	32.8		8.68 (6.31 - 11.95)
**Comprehensive practice**					
Good practice	576	30.6	69.4	<0.001	1
Bad practice	282	76.6	23.4		7.44 (5.36 - 10.32)

**Association of socio-demographic characteristics and comprehensive knowledge, attitude and practice with prevalence of cautery**: the odds of practicing cauterization among respondents aged 40-59 years was increased by 65% compared to those aged 18-39 years (AOR=1.65, 95%CI 1.23-2.21). Moreover, the probabilities of practicing cauterization was found to be 2.3 times more among respondents aged 60 and above compared to respondents aged 18-39(AOR=2.27, 95%CI 1.46-3.53). Muslims are more prone to practice traditional cautery as compared to Christians (AOR=12.42, 95% CI: 6.59-23.41). Further, compared to respondents with no education the odds of cauterization was reduced by 46% and 78% among primary (AOR=0.54; 95% CI: 0.38 - 0.77) and middle and above (AOR=0.22; 95% CI: 0.15 - 0.31) respectively (Table 3). Those ever married were found to be two times more likely to practice traditional cautery (AOR=2.3, 95% CI: 1.53 - 3.46) as compared to the single ones. Also, patients who have poor access to health facility were practicing traditional cautery about 7 times more than those who had good access to nearby health facility (AOR=6.9, 95%CI 3.46-13.75). Traditional cauterization was also commonly practiced by the ethnic groups of Afar (55.3%), Saho (50.6%), Tigre (50.5%) and Rashaida (68.5%). Except in Tigrigna (6.5%) ethnic group, the practice was prevalent among the other ethnic groups with the highest practice observed in the Rashaida ethnic group (AOR=31.44, 95%CI 12.95-76.30).

Study participants who had poor knowledge were practicing cautery about six times higher when compared to those who had good knowledge (AOR=6.45, 95% CI: 4.69 - 8.87). Patients with poor attitude were found to be about nine times more to practice cautery (AOR=8.68, 95% CI: 6.31-11.95). Whereas, respondents with bad practice have seven times higher prevalence of practicing traditional cauterization when compared to their counterparts (AOR=7.44, 95% CI: 5.36-10.32) and were more frequently experiencing cautery at least once in their life (Table 3).

## Discussion

This study revealed that the self-reported practice of traditional cauterization in patients visiting Massawa hospital was found to be prevalent (45.7%). This is higher than the study conducted in Muslim Bedouin patients who came to clinics in southern Israel [8], which account to, 35.7%. The higher prevalence of traditional cauterization in this study could possibly be due to inadequate awareness of the community as about half of the study participants were illiterate and had limited access to primary healthcare services mainly due to poor or unavailability of transportation services. Though 73% of the study population had access to healthcare services within 10 kilometers, transportation service was identified as a significant problem in majority of the respondents. Cultural and religious believes, the availability and popularity of high number of traditional healers in the community, and the belief of the population that traditional cautery is effective could be additional factors for the high prevalence of cautery among the study participants.

The practice was common among Muslims and one possible explanation is that the Muslims might have higher cultural or traditional acceptance towards traditional cautery compared to the Christians. This might also be attributed with the fact that majority of the Muslim participants had poor knowledge, attitude and practice on traditional cauterization. The practice of cautery was higher on patients who had no access to health facility. Except in the Tigrigna ethnic group, the practice of cautery was found to be common in all of the ethnicities available in the region. Based on [10], cauterization was a widely used traditional practice in Arabic countries among both pediatric and adult patients. Again, this is similar with the aforementioned explanation as all these ethnicities that commonly practice cautery are Muslims and almost all the Tigrigna ethnic group were Christians. In this study, majority of the participants (95.3%) were familiar with traditional cautery which is much higher than reported in a similar study conducted in Israel [8]. Nine out of ten of the study participants knew someone that undertook cautery in their community or family members, which is also much higher than reported elsewhere (62.4%), [8]. This could be due to the high illiteracy rate, higher cultural/religious beliefs and the high popularity of traditional healers in the community.

Upper extremities, head, lower extremities and abdomen were the commonly reported body parts cauterized. Metal rod was the main cauterant used followed by wood/thorn and knife. Almost half of the respondents reported that traditional healer´s use same cauterant for more than one person, however, several respondents did not know whether the cauterant was sterilized or not. While, the self-reported sterilized cauterant were treated mainly with fire and some with boiled water, this showed that patients were cauterized either with unsterilized or poorly treated tools for cauterization which puts patients at risk of communicable diseases such as HIV and hepatitis apart from its potential complications. The main indication of cauterization reported was to treat hepatitis followed by joint inflammation and evil eye. If hepatitis was really present at least in some of the cases, the risk of transmission is even higher. The belief that cauterization is effective against hepatitis was also reported in the Israeli study [8]. The fact that almost half of the study participants understood that traditional cautery does not transmit communicable diseases reflect poor awareness of the community which urges immediate intervention. Traditional cautery was more commonly reported in elderly compared to other age groups; as the age of the patients increases, the practice of cautery significantly increases, which is similar to findings of other studies [8,11]. Those older age group also had poor attitude and showed poor knowledge. The influence of education on traditional cautery was evident, because as the level of formal education increased, there was a significant decrease of the practice.

The belief that cautery can treat diseases that cannot be cured by conventional medicine, its effectiveness and safety, religious/cultural conviction and its affordability compared to conventional medicine were the main reasons reported for practicing traditional cautery. Despite the aforementioned impression of the study population on traditional cautery and a high satisfaction rate reported, almost three-fourth of them declared that they have no plan to practice it in the future. Similarly, majority of the respondents did not encourage others to practice cautery. Taking the above-mentioned facts that favors cautery into consideration, it is difficult to comprehend why majority of the participants were planning to refrain from traditional cautery and only less than a quarter are planning to do it in the future. This requires further qualitative studies to identify the reasons behind. About three-fourth of the study participants disclosed that they did not inform healthcare professionals, about their history of traditional cautery, during their visit to health facilities. Majority of those who did cautery in past followed their relatives´ advice; whilst in a study conducted in Libya, it was reported as 90% [12]. Of these who practiced cautery, self-reported improvement was substantial, similar to reported in the Israeli study [8]. Moreover about one in ten patients encountered treatment complications in this study while, according to [12], 63.5% of participants did not improve after cauterization and developed adverse events and complications. The most common complications were body disfigurement and infection. It is not understood how traditional cautery played a role in improving disease conditions; but might be explained by pain shift.

**Limitations**: lack of qualitative data could not explore the why and how questions raised with this quantitative study. Besides, the prevalence of traditional cautery among the study participants was self-reported and thus, might be under-estimated. Hence, the study was conducted in a specific geographic region which lacks generalizability to the Eritrean population. Health facility-based interview might also limit participants to openly express their attitude and practices.

## Conclusion

Traditional cauterization in patients attending Massawa hospital of the Northern Red Sea Zone was found to be prevalent. Traditional cauterization was more prevalent and significantly associated on those with poor knowledge, poor attitude and bad practice. Traditional cautery was also common in older age group and illiterate patients who have poor access to health facility. The prevalent practice of cautery with unsterilized tools and reported complication in several patients warrant immediate attention from policy makers. Qualitative and larger studies are required to further explore the basis for the frequent use of cautery if there are genuine effects for cautery on the human body in the community. Encouraging healthcare professionals to report cauterization related complications, raising public awareness through mass medias and health facilities for safety concerns and complications related to cauterization, developing legal framework and code of ethics on traditional medicine in general and development of a proclamation that instruct the termination of traditional cautery are among the recommended strategies to protect the public health.

### What is known about this topic

- Traditional practitioners believe that cautery prevent or treat diseases;
- Studies revealed it causes severe infections and complications, physical deformities and death;
- Significant portion of the population living in the developing countries practice traditional cautery.

### What this study adds

- The practice was prevalent in patients visiting Massawa hospital, Eritrea;
- Cautery was much higher in the Muslim participants than the Christians;
- Highly associated in these with poor knowledge, poor attitude and limited access to health facilities of the patients.
